# Development and Application of Stable Phantoms for the Evaluation of Photoacoustic Imaging Instruments

**DOI:** 10.1371/journal.pone.0075533

**Published:** 2013-09-25

**Authors:** Sarah E. Bohndiek, Sandhya Bodapati, Dominique Van De Sompel, Sri-Rajasekhar Kothapalli, Sanjiv S. Gambhir

**Affiliations:** Bio-X Program and Department of Radiology, Molecular Imaging Program at Stanford, Stanford University School of Medicine, Stanford, California, United States of America; University of California, Irvine, United States of America

## Abstract

Photoacoustic imaging combines the high contrast of optical imaging with the spatial resolution and penetration depth of ultrasound. This technique holds tremendous potential for imaging in small animals and importantly, is clinically translatable. At present, there is no accepted standard physical phantom that can be used to provide routine quality control and performance evaluation of photoacoustic imaging instruments. With the growing popularity of the technique and the advent of several commercial small animal imaging systems, it is important to develop a strategy for assessment of such instruments. Here, we developed a protocol for fabrication of physical phantoms for photoacoustic imaging from polyvinyl chloride plastisol (PVCP). Using this material, we designed and constructed a range of phantoms by tuning the optical properties of the background matrix and embedding spherical absorbing targets of the same material at different depths. We created specific designs to enable: routine quality control; the testing of robustness of photoacoustic signals as a function of background; and the evaluation of the maximum imaging depth available. Furthermore, we demonstrated that we could, for the first time, evaluate two small animal photoacoustic imaging systems with distinctly different light delivery, ultrasound imaging geometries and center frequencies, using stable physical phantoms and directly compare the results from both systems.

## Introduction

Photoacoustic imaging is an emerging modality that exploits the photoacoustic effect to combine the high contrast of optical imaging with the spatial resolution and penetration depth of ultrasound. The technique relies on the absorption of nanosecond laser pulses by tissue chromophores (either endogenous, or administered molecular imaging agent(s)), which produce an increase in temperature and consequently a thermoelastic expansion; after propagating through the tissue, the broadband acoustic waves are detected using ultrasound receivers and images of the absorbed optical energy density can be reconstructed.

The main advantage of this hybrid approach is that the optical properties of biological tissue, including high contrast and spectral specificity, are encoded in an ultrasound signal. Since acoustic waves are scattered far less than photons in tissue, photoacoustic signals can be detected at far greater depths than traditional optical imaging techniques, with depths of up to 7 cm reported in living subjects [1]. Our laboratory is currently pursuing photoacoustic molecular imaging for application in small animals, using both bespoke and commercial preclinical imaging devices, combined with novel contrast agents [2,3,4], and is actively involved in clinical translation of the technique, using a transrectal endoscopic photoacoustic device for prostate cancer diagnosis [5].

The major limitation currently facing these studies is the lack of a robust, reliable and stable phantom for quality control and evaluating the performance of different photoacoustic imaging instruments before embarking on animal or human studies. Physical phantoms for photoacoustic imaging are needed to [6]:

- Perform routine quality control to understand reproducibility over time and between laboratories
- Validate performance of reconstruction algorithms and data corrections
- Optimize the imaging signal-to-noise ratio for a given application
- Compare the detection limits and spectral accuracy of different system designs
- Understand the maximum achievable depth of penetration for *in* *vivo* imaging
- Translate our knowledge and experience from the preclinical to the clinical setting

The purpose of this study was to design, fabricate and evaluate a stable physical phantom that would enable us to begin addressing these points. Given the wide range of optical excitation wavelengths and transducer frequencies employed for photoacoustic imaging, as well as the biological range of optical absorption coefficients, optical scattering coefficients, speed of sound and ultrasound attenuation, developing a characterization standard for this modality is challenging. We therefore set a number of design constraints upon our physical phantom. Firstly, both the targets and matrix should be composed of the same material, avoiding a mismatch of acoustic, mechanical or thermoelastic properties within the phantom [7,8]. Secondly, the targets should be of a defined shape, size, position and provide optical absorption similar to our biological imaging targets. Thirdly, it should be possible to tailor both the optical and acoustic properties of background matrix, in which the targets are embedded. In particular, the phantom should resemble biological tissue in the near infrared wavelength range of 680 nm-950 nm [6,9,10]. Finally, the phantom should provide: a stable signal as a function of time; be resistant to physical degradation or bacterial invasion; and be easy to prepare and handle.

Numerous materials have been reported with suitable properties for use in both optical and ultrasound phantoms; for an extensive discussion, readers are referred to the excellent reviews of Pogue and Patterson [6] and Culjat et al. [9] respectively. Hydrogels, including agarose and gelatin, are the most widely reported bulk matrix materials. For photoacoustic imaging, the addition of india ink, whole blood, copper/nickel chloride and fluorescent dyes have been reported to provide optical absorption [11,12,13]. Intralipid and titanium oxide have been used to tailor optical scattering, while spherical silica particles have been added to adjust acoustic backscattering [12,14]. However, hydrogels will absorb the water used for ultrasound coupling, suffer from dehydration and bacterial growth in storage over time and are highly susceptible to physical damage [9,15,16]. Although it is possible to extend the longevity of these materials by careful storage and addition of chemicals such as formaldehyde, this adds greater complexity to both the phantom fabrication and long-term application. Furthermore, water soluble dyes diffuse in these gels, so defined target shapes require encapsulation for example in PMMA tubing, creating an acoustic boundary.

For increased stability and inter-laboratory comparisons of optical imaging techniques, alternative materials have emerged that represent a potential compromise between longevity and tissue mimicking features for photoacoustic imaging. Polyvinyl alcohol (PVA), a synthetic polymer cryogel, has been demonstrated as a phantom material for photoacoustic mammography [17,18,19]. PVA has greater longevity and structural rigidity compared to hydrogels, but requires extensive preparation including freeze-thaw cycles lasting several days and may be sensitive to humidity [6]. Polyvinyl chloride plastisol (PVCP) is an oil-based material insoluble in water that polymerizes and becomes translucent when heated to high temperatures. Preliminary material validation of PVCP was performed by Spirou et al. [20]. While PVCP has been used in a small number of other studies [21,22], it has yet to be fully exploited as a phantom material for photoacoustic imaging.

The overall aim of this work was to design and test a stable physical phantom for photoacoustic imaging that would enable routine quality control and quantitative evaluation of the performance of different imaging systems. Having experimented with the range of materials described above, we selected PVCP as the phantom material that provided the optimum solution to our design constraints. Our simple fabrication procedure allows us to tailor both the optical absorption and scattering properties of the matrix material to values relevant for studies of biological tissue in the near infrared.

Using this knowledge, we designed and constructed three different types of phantom to optimize and evaluate two commercial photoacoustic systems designed for small animal imaging that are in routine use at our institution. The VisualSonics Vevo LAZR integrates laser excitation with a high frequency VisualSonics linear array transducer, enabling real time cross sectional (B-mode) micro-ultrasound and photoacoustic imaging in the same plane. A linear scan of the transducer provides 3D volumetric imaging. The Endra Nexus 128 is a tomographic instrument, providing inherently 3D reconstructions of the photoacoustic signals from a sample using a helical array of unfocused transducers. Our phantom measurements included: evaluating reproducibility of the imaging data over time (routine quality control); optimizing the energy compensation algorithm; defining the linearity of the photoacoustic response; tuning the imaging parameters to maximize the signal-to-noise ratio and evaluating the limits of detection in the presence of optically absorbing and scattering background. Our results show that PVCP phantoms are easy to design and fabricate, and provide a robust and reliable method for quantifying the performance of different photoacoustic imaging instruments.

## Materials and Methods

### 2.1: Phantom design and construction

#### 2.1.1: General design considerations

The “standard” polyvinyl chloride plastisol (PVCP; M-F Manufacturing Co., Fort Worth, TX, USA) preparation is optically transparent and provides a density and acoustic parameters equivalent to water [20,23]. The speed of sound of PVCP (1,400 ms^-1^) is around 15% lower than the soft tissue average, but since our goal in this study is to develop a stable phantom for quality control and system evaluation, this was not considered to be a significant limitation. Furthermore, it has previously been shown that the acoustic properties of PVCP can be modified by the addition of plastic hardener and softener, enabling the speed of sound and elasticity to be tuned [23]. It would therefore be possible to better match the properties of a particular organ of interest if needed. In this study, we have focused exclusively on modulating the photoacoustic signal generation by changing the optical properties of the phantom. Establishing detection limits by modulating the ultrasound properties of the PVCP within the bounds of realistic soft tissue averages is the subject of ongoing work.

To satisfy our design constraint of using the same material for both the target and background in the phantom, we attempted to embed absorbing shapes fabricated using PVCP into layers within phantoms. Spheres provide an optimal photoacoustic target for testing data reconstructions, especially in tomographic imaging systems. We found that we could repeatably mold PVCP into spherical targets with a range of sizes and defined optical absorption properties then embed them in the background matrix without any distortion of the shape or diffusion of the absorbing dye.

#### 2.1.2: Materials

The optical properties of our phantoms were defined by adding black plastic color (BPC; M-F Manufacturing Co., Fort Worth, TX, USA) to adjust the absorption coefficient *μ*_*a*_ and titanium oxide (TiO_2_) powder (232033; Sigma-Aldrich, St. Louis, MO, USA) to adjust the reduced scattering coefficient$\mu_{S}^{'}$. We selected the ranges for *μ*_*a*_ and $\mu_{S}^{'}$ based on typical literature values for mammalian tissue in the wavelength range 600-1000 nm of *μ*_*a*_~ 0.05-10 cm^-1^ (0.1-1 cm^-1^ average) and $\mu_{S}^{'}$~ 2-50 cm^-1^ (5-10 cm^-1^ average) [10,24,25]. The absorbing properties of BPC are provided by carbon black dye (CAS 1333-86-4) and were independently measured using BPC dissolved in DMSO using a spectrophotometer (Evolution 60, Thermo Scientific). The absorption measured in PVCP and DMSO were directly related for the range of concentrations used in this study (linear fit slope 1.08 ± 0.06) ; measurements were made in DMSO due to ease of preparation. The concentration range used in this work for spherical targets is 0.064-0.256% volume fraction (v/v), corresponding to *μ*_*a*_ = 0.26-1.07 (± 0.01) cm^-1^ between 600-1000 nm, while for background absorption the range is *μ*_*a*_ = 0-0.1 (± 0.01) cm^-1^(0-0.016% v/v). Dynamic light scattering was performed on samples of TiO_2_ powder dissolved in water, which confirmed the size of particulates to be < 450 nm. The concentration range used for background materials in this work (TiO_2_ was not included in targets) is 0-2.5 mg/ml. The reduced scattering coefficient for this range of TiO_2_ concentrations was measured using the method of Akarçay et al. [26] to be $\mu_{S}^{'}$ ~ 0.9-6.8 cm^-1^ at 750 nm, in good agreement with the range found in previous studies [20,22,27,28]. The ultrasound attenuation of PVCP does not change with the addition of TiO_2_ [20].

#### 2.1.3: Preparation of background matrix and spherical targets

**Figure 1:**
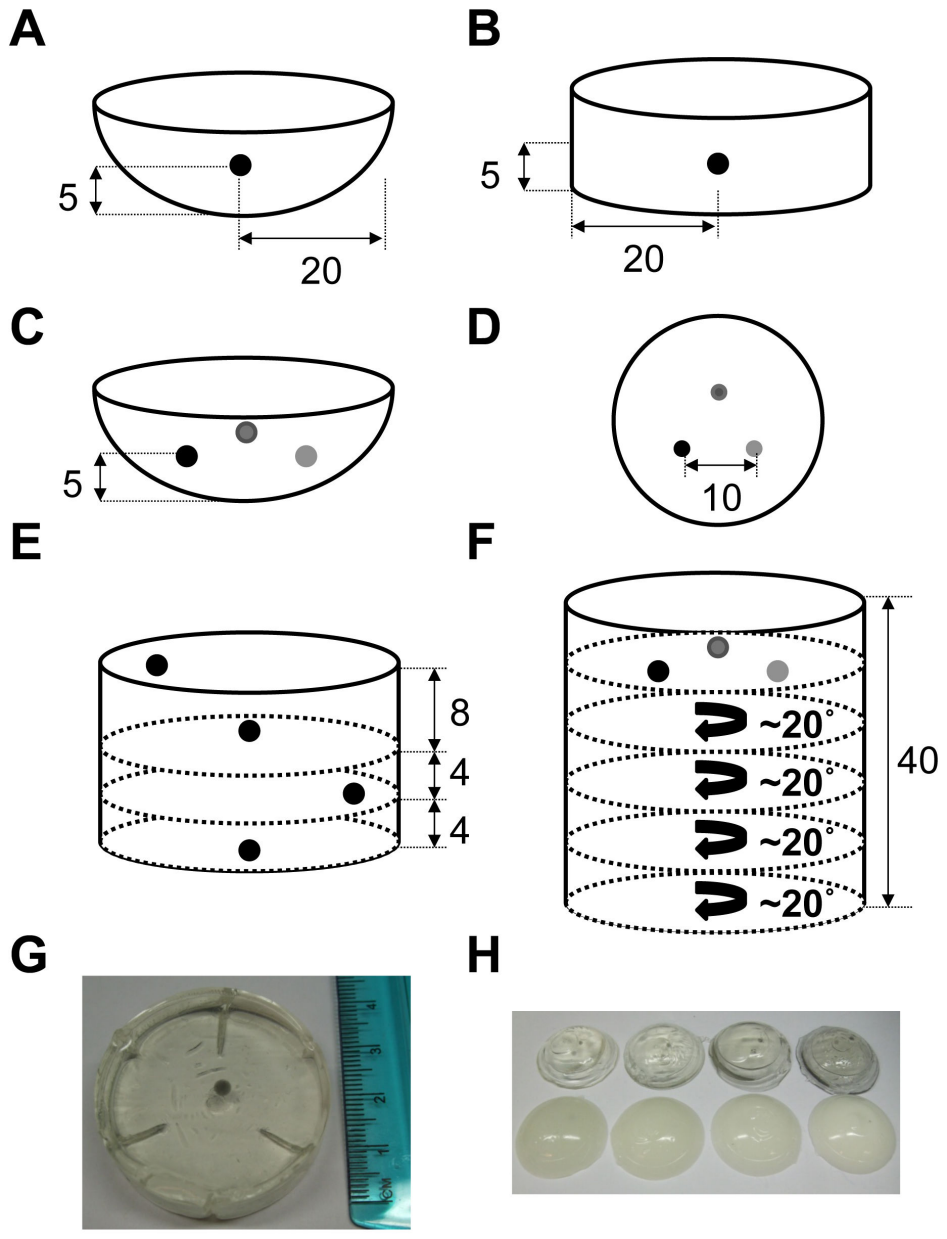
Illustrations and photographs of the physical phantoms designed for evaluation of photoacoustic imaging systems. Quality control phantoms (P1 in Table 1) were composed of a single spherical target embedded at 5 mm depth in an optically transparent matrix. For the Endra system, a hemispherical phantom was fabricated (A) and imaged from below. For the VisualSonics system, a cylindrical phantom was fabricated and imaged from above (B; photograph shown in G). Two separate phantoms were made to be kept next to the imaging system and routinely imaged. To assess the effect of optical absorption and scattering on target visibility, a range of phantoms were made that contained three spheres with different optical absorption, as well as different background absorption and scattering properties (C, side view; D, top view). The photograph in H, from the top left, shows the phantoms where the background matrix has an increasing optical absorption (upper line) or scattering (lower line). Two phantoms were designed to assess the depth of penetration of the systems, the first with a strongly absorbing sphere embedded at four different depths up to 16 mm (E) and the second with a range of spherical targets embedded up to 40 mm (F). The pattern of spheres was rotated by an angle of approximately 20 degrees between layers to avoid absorption by more superficial spheres affecting the signal intensity from the deeper spheres. All dimensions are in mm.

All phantoms were prepared according to protocol S1. Briefly, the PVCP was heated under vacuum (Figure 1A) until the phase transition to a translucent liquid was complete after 10 minutes. The solution was then poured into the chosen mold within 1-2 minutes and it solidified within 5 minutes. The range of optical absorption and scattering was achieved by serial dilution from a stock solution at the highest concentration, to minimize errors in preparation. Spherical targets were formed using a bespoke aluminum mold in two parts, which contained hemispherical indentations (available diameters: 2, 2.8 and 3.2 mm) on each side and guiding posts for accurate alignment [29] (Figure 1B). Targets were positioned at a fixed depth by pouring consecutive layers of PVCP background material. Each layer was allowed to cool partially before the spherical targets were placed and the next layer was poured. The total time for phantom preparation ranged from 30 minutes to 2 hours. These phantoms have remained stable, without degradation in shape, size or sign of bacterial or other growth for over 6 months when stored in air tight containers at room temperature. The phantom designs for routine quality control and system performance evaluation are shown in Figure 1 and listed in Table 1, with further details given below.

**Table 1:** List of physical phantoms constructed and their design parameters.

**Phantom Name**	**Bkgd Absorption (% BPC)**	**Bkgd Scattering (mg/ml TiO_2_)**	**Target Absorption (% BPC)**	**Target Size (mm)**	**Target Depth (mm)**
**P1 QC**	0	0	0.256	3.2	5
**P2 Bkgd Absorb**	0, 0.004, 0.008, 0.016	0	0.064, 0.128, 0.256	2	5
**P3 Bkgd Scatter**	0	0.25, 0.5, 1, 2.5	0.064, 0.128, 0.256	2	5
**P4 Depth**	0.004	0.5	0.256	2.8	0, 8, 12, 16
**P5 Depth**	0.004	0.5	0.064, 0.128, 0.256	2.8	9.1, 15.3, 17.3, 23.3, 35.5

The phantoms were fabricated with varying target sizes, depths and absorption. Values are quoted by material properties rather than optical absorption or scattering coefficients, as these will vary as a function of wavelength. P1 refers to the phantoms shown in Figure 1A and 1B. P2 and P3 refer to a series of phantoms with the different background properties listed, as shown in Figure 1C and 1D. The P4 and P5 depth phantoms are illustrated in Figure 1E and 1F. Abbreviations: BPC = black plastic color; TiO_2_ = titanium oxide; Bkgd = background; QC = quality control.

#### 2.1.4: Routine quality control phantom (P1)

Our quality control phantom was designed to enable: validation of our data reconstruction and corrections; establish the reproducibility of the imaging systems over time; and to aid in optimizing the imaging signal-to-noise ratio. These phantoms were composed of a single highly absorbing target (0.256% BPC, 3.2 mm diameter) embedded at 5 mm below the surface. We decided to construct two phantoms with the same properties but different geometries: one with a hemispherical shape (Figure 1A), for imaging in the tomographic system under test and one with a cylindrical shape (Figure 1B and photograph in Figure 1G), appropriate for the linear array system. The fabrication of two quality control phantoms allowed them to be kept with the system at all times and imaged at the start of each set of measurements to confirm system performance.

#### 2.1.5: Robustness phantoms (P2 and P3)

These phantoms were designed to test the robustness of the photoacoustic signals measured in a background of changing optical properties. A range of TiO_2_ and BPC concentrations were added to the background PVCP preparation and sonicated at 40 °C for 10 minutes prior to heating. This ensured uniform distribution of the scattering particles and absorbing medium throughout the phantom. Three spherical targets of 2.8 mm diameter, with 0.064, 0.128 and 0.256% v/v BPC concentrations respectively, were embedded at 5 mm depth from the bottom of the hemisphere and at 5 mm depth from the planar top surface. The phantoms contained increasing concentrations of absorbing (P2 series in Table 1) and scattering (P3 series in Table 1) media, within the range encountered in soft tissue. The layout of the targets is shown in Figure 1C and 1D, while the photograph in Figure 1H illustrates the constructed phantoms. The same phantoms were imaged on both systems, with an additional holder constructed to mount the hemispherical phantoms into the VisualSonics system, to enable imaging from above (i.e. directed onto the planar surface).

#### 2.1.6: Depth phantoms (P4 and P5)

To understand the maximum achievable depth of penetration for *in vivo* imaging, two phantoms were fabricated with fixed background optical properties (minimally absorbing and scattering with 0.004% v/v BPC and 0.5 mg/ml TiO_2_) but with the three concentrations of absorbing targets positioned at different depths up to a maximum of 16 mm in phantom P4 and 35 mm in phantom P5 (Figure 1E and 1F). The pattern of spheres shown in Figure 1F was rotated by an angle of approximately 20 degrees between successive layers to avoid absorption by more superficial spheres affecting the light delivery to deeper spheres. Again, the same phantoms were imaged on both systems.

### 2.2: Photoacoustic Imaging of the Phantoms

#### 2.2.1: VisualSonics Vevo LAZR

**Figure 2:**
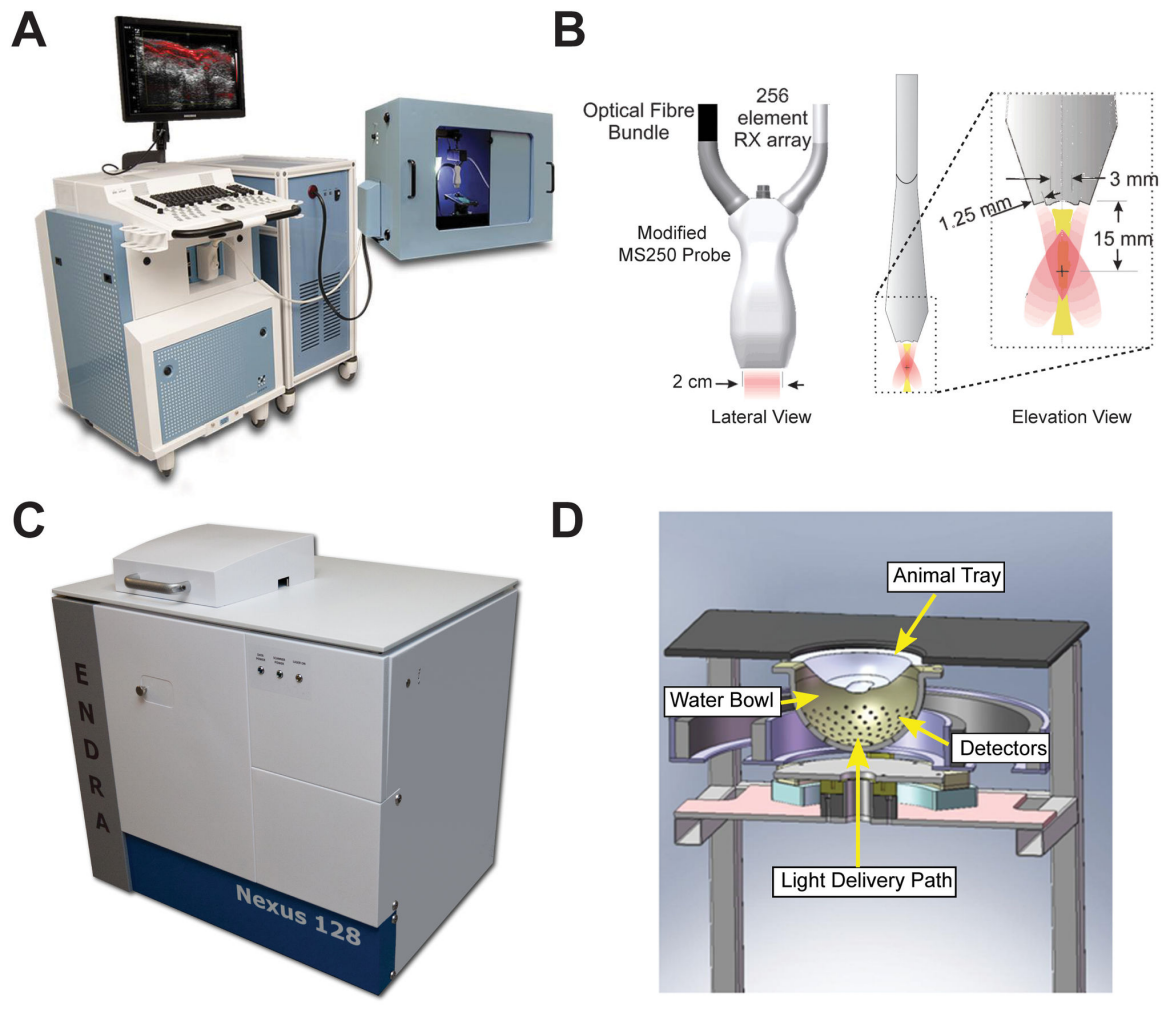
Schematics of experimental systems used. (A) The VisualSonics Vevo LAZR uses intersecting planar laser beams (B) for excitation and a linear transducer array for 2D imaging of the resulting ultrasound. (C) The Endra Nexus 128 delivers diffuse laser light and detects the ultrasound using 128 transducers in a helical arrangement (D) for 3D reconstruction.

An illustration of the VisualSonics Vevo LAZR system (referred to as VisualSonics for the remainder of the article) and a schematic of the LAZR transducer are shown in Figure 2A and 2B. A full description of the system design is available elsewhere [30]. Briefly, photoacoustic signals were excited by a pulsed (20 Hz, < 10 ns pulse width) and tunable (680-970 nm) Nd:YAG laser (OPOTEK Inc., Carlsbad, CA, USA) used to illuminate the sample through two rectangular fiber optic bundles placed on either side of a linear array transducer (MS-250, center frequency = 21 MHz, 256 elements) at an angle of 30° to the imaging plane. For each laser pulse, photoacoustic signals were captured on a quarter of the transducer array (64 elements), then once all quarters were acquired, the full dataset was used to beam form a single image plane. Acquisition was extended to 3D by linearly translating the imaging plane with a stepper motor. Photoacoustic signals were acquired with a laser surface fluence of < 20 mJ/cm^2^ and displayed at 5 Hz.

**Figure 3:**
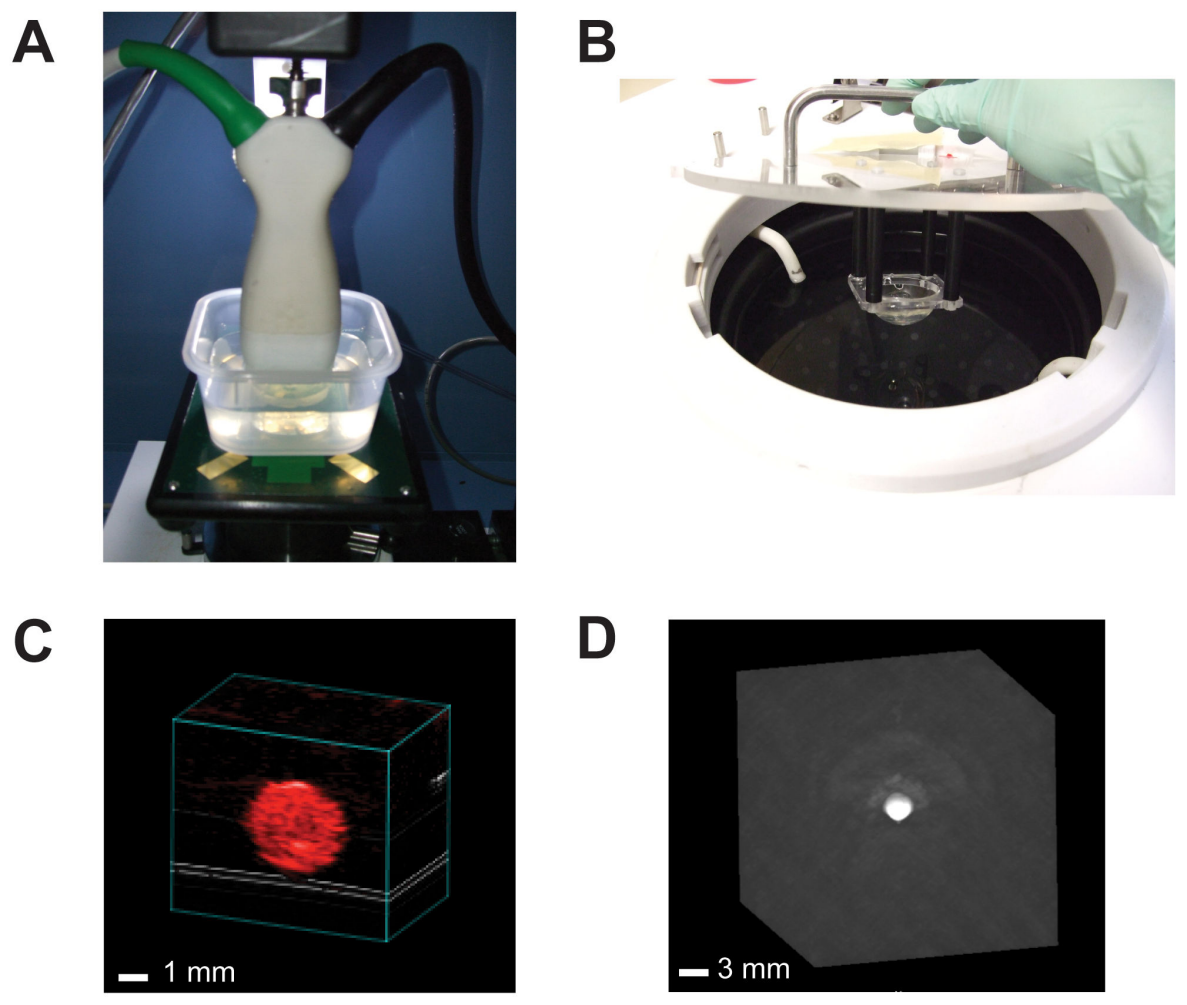
Placement of the quality control phantoms into the imaging systems and example images. Phantoms were positioned in VisualSonics Vevo LAZR (A) and Endra Nexus 128 (B) using water for ultrasound coupling. A simple acrylic box was used to position the phantom from Figure 1B in the VisualSonics system, while the hemispherical mold that was used to make Figure 1A was placed directly into the Endra system. (C) Example of a cross section of the quality control phantom on the VisualSonics system. (D) Example of a tomographic reconstruction of the same phantom from the Endra system.

The phantom to be imaged was placed in a custom acrylic holder and water coupled to the transducer (Figure 3A); targets placed at 5 mm depth within the quality control phantom were always positioned at 10 mm below the ultrasound transducer, which is set back behind the fiber optic bundles (see Figure 2B). The experimental setup for phantom imaging is as similar as possible to that used during animal imaging. The laser energy delivered by the system was monitored via a beam splitter in the light path throughout the experiment and we also measured the energy using an external power meter (Maestro, Gentec, Lake Oswego, OR, USA) placed at the transducer head at the beginning and end of each experiment. System parameters varied in the imaging optimization process included gain and persistence (frame averaging).

#### 2.2.2: Endra Nexus 128

The Endra Nexus system (referred to as Endra for the remainder of the article) schematic is shown in Figure 2C and 2D; the full system design can be found in Kruger et al. [8]. The detection system consists of an array of 128 individual, unfocused transducers (center frequency = 5 MHz) positioned in a spiral pattern on a hemispherical surface with a 101 mm radius of curvature. Laser light, provided again by a tunable (680-950 nm), pulsed (7 ns pulse width) Nd:YAG laser (OPOTEK Inc., Carlsbad, CA, USA), was delivered from below via a planoconvex lens at the bottom of the hemisphere and provided a light beam of approximately 20 mm in diameter at the sample. The phantom to be imaged was placed in a custom, hemispherical acrylic holder (also used to mold the phantom shape) and suspended in the center of the bowl array (Figure 3B). The transducers are coupled to the sample plane by filling the bowl with water, which is maintained at 38°C by a pumping system.

Photoacoustic signals generated by a given laser pulse in a target at 5 mm depth in a phantom are detected by all transducers, after travelling through ~0.5 cm within the phantom and ~8 cm in water. The experimental setup is similar to that used during animal imaging. Rotating the hemispherical bowl to multiple angular positions increases the density of k-space sampling, while maintaining uniform angular sampling. Multiple laser pulses and angular positions can be used to increase the signal-to-noise ratio (SNR, see definition in Section 2.3) in a single image volume at the expense of acquisition time. The laser energy delivered by the system was monitored via a beam splitter in the light path throughout the experiment and an average value recorded for each angular view. The imaging optimization study performed on this instrument tested directly the effect of increasing the number of views and laser pulses on the improvement in SNR and allowed this to be traded off against scan time. In all multiwavelength imaging, fewer data points were acquired with the Endra system in order to keep measurement times practical.

### 2.3: Image Reconstruction and Data Analysis

All data in this work were acquired and analyzed both with and without the built-in energy compensation methods offered by both vendors in order to assess their accuracy. All VisualSonics Vevo LAZR data was beamformed using a delay and sum algorithm [30] and analyzed using the region of interest (ROI) drawing tools in the dedicated VisualSonics software package (v1.4 and prototype v1.5 for 3D regions; VisualSonics, Toronto, Canada), which provided the mean photoacoustic signal within the ROI. As will be described, using our quality control phantom we found that the built-in algorithm for energy compensation in the VisualSonics system, which used the energy measurement from the beam splitter described in Section 2.2.1, did not accurately correct for the energy delivered to the sample. At the time of writing, the manufacturer did not provide an external calibration option, therefore we implemented a manual correction for all our remaining measurements based on our record of the laser energy at the output of the LAZR transducer head. This correction was found to yield a flat spectrum for BPC absorbing targets and hence was considered a more appropriate energy correction. Based on this knowledge, an external calibration method is now available for the LAZR instrument.

Endra Nexus 128 data was reconstructed in volumes of 256 x 256 x 256 with 0.1 x 0.1 x 0.1 mm^3^ voxels using their filtered back projection algorithm (reconstruction time < 1 minute). The difference in the speed of sound between water and PVCP was accounted for in the reconstruction by adjusting a single overall speed of sound to achieve maximum focus, and energy compensation was performed on a per view basis. Analysis in 2D was performed on image maximum intensity projections (MIPs) in the Fiji distribution of ImageJ (NIH, Bethesda, USA), while 3D volumes were drawn in Osirix after exporting the raw data to DICOM format (Pixmeo, Switzerland).

For both systems, the following metrics were derived for all image data: mean target ROI signal, mean background ROI signal, signal-to-background ratio (SBR, the ratio of mean target signal to the mean background signal) and signal-to-noise ratio (SNR, the ratio of mean signal to standard deviation of background). Data are shown as mean ± standard error unless otherwise stated and least squares curve fitting on data from regions of interest was performed in Prism (Graphpad, La Jolla, CA, USA).

## Results

Having established methods for the design and fabrication of our physical phantoms for photoacoustic imaging (see Section 2.1 and protocol S1), we then used the resulting phantoms to aid routine quality control and quantitative evaluation of performance in the two small animal imaging instruments shown in Figure 2.

### 3.1: Photoacoustic signal stability as a function of wavelength, concentration and time measured using the quality control phantom

The spherical target in the quality control phantom P1 containing 0.256% v/v BPC (*μ*_*a*_= 1.07 ± 0.01 cm^-1^) was visible with clearly defined boundaries on both systems across all available wavelengths, indicating accurate reconstruction of the absorbed optical energy density using the methods described in Section 2.3; example images acquired at 750 nm are shown in Figure 3C and 3D. As expected, no evidence of an acoustic boundary is present in these images, however, the smoothness of the boundary combined with a relatively low concentration of absorbers means that photoacoustic speckle can be observed within the spherical targets [31,32]. This phantom was used to assess the stability of the photoacoustic imaging measurements as a function of illumination wavelength, BPC dye concentration and time.

**Figure 4:**
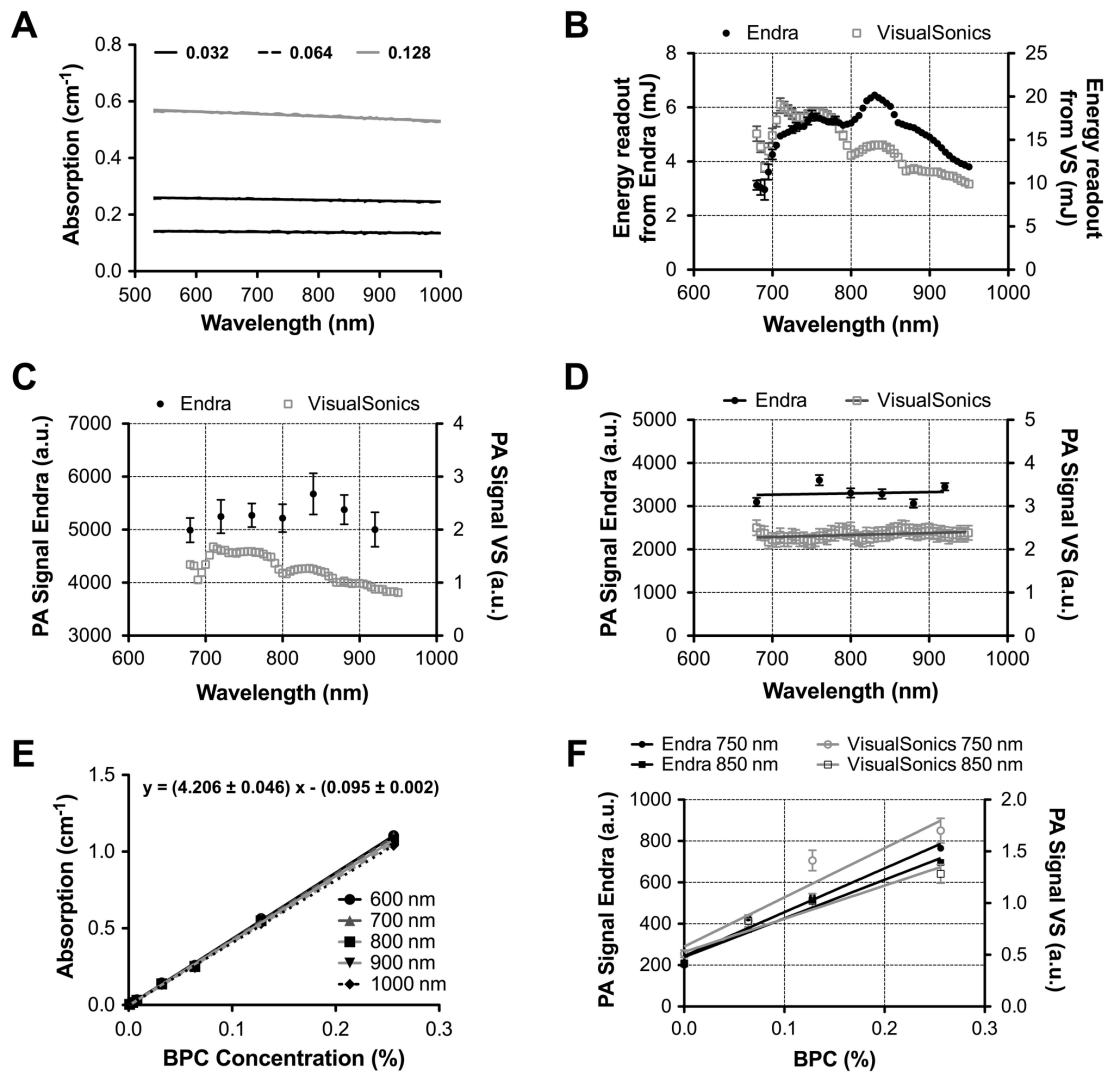
Photoacoustic imaging performance assessed using a PVCP quality control phantom in both small animal imaging instruments. (A) Absorption spectrum of the black plastic color (BPC) dye at three concentrations (0.032%, 0.064% and 0.128%) recorded on a spectrophotometer (B) Spectrum of laser energy as a function of wavelength recorded by an external power meter. (C) PA signal recorded from the target sphere without energy compensation. (D) PA signal recorded from target sphere once normalized for recorded energy per view (Endra) or per image (VisualSonics). (E) Linearity of BPC absorption as a function of concentration at five different wavelengths. (F) Linearity of PA signal recorded as a function of increasing BPC concentration at two different wavelengths.

The absorption spectrum of the BPC dye (as measured independently on a spectrophotometer) is shown in Figure 4A and the laser energy as measured at the photoacoustic imaging system output is shown as a function of wavelength in Figure 4B. Despite using similar laser sources, there is a distinct difference in the spectral output from the VisualSonics and Endra systems, explained by the different light delivery paths (see Figure 2) from source to sample. The photoacoustic signals recorded from the spherical target as a function of wavelength are shown both without (Figure 4C) and with (Figure 4D) the energy compensation. A linear fit to the data in Figure 4D should have zero slope (within an error of 6%) based on the spectrum of the BPC dye in Figure 4A. It should be noted that these measurements were instrumental in selecting the method of energy compensation, as initial attempts to correct the VisualSonics data using the built-in algorithm produced erroneous results. After appropriate energy compensation (see section 2.3), slopes of photoacoustic signal as a function of wavelength were 0.00046 ± 0.0013 nm^-1^ and 0.296 ± 1.182 nm^-1^ (neither significantly non-zero, p=0.61 and 0.81) on VisualSonics and Endra respectively. The larger error observed in the Endra data reflects the fact that fewer data points were acquired due to the longer scan duration. Figure 4E shows that there is a linear increase absorption of the BPC dye as a function of concentration as measured with a spectrophotometer. Figure 4F illustrates that both systems show a linear response as a function of BPC dye concentration for the wavelength range under investigation (680nm-950nm, data shown only from 750 nm and 850 nm for clarity), up to the maximum value used in this study (0.256% v/v, *μ*_*a*_= 1.07 ± 0.01 cm^-1^).

**Figure 5:**
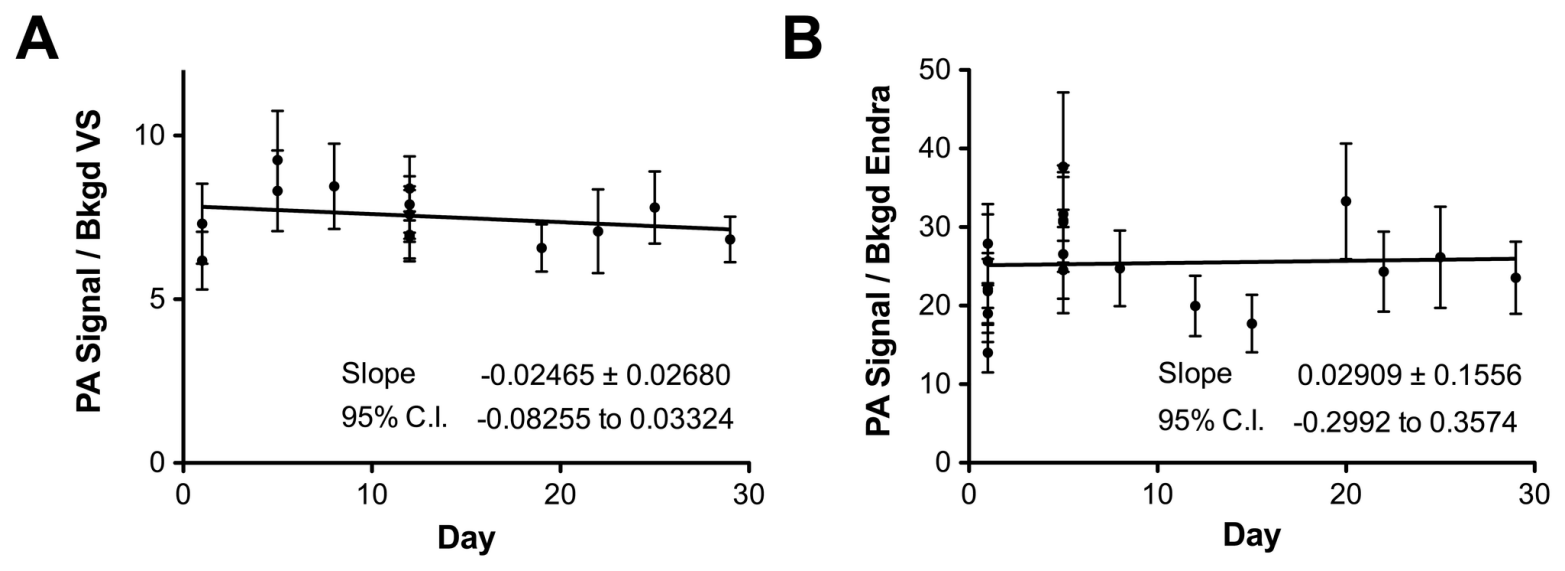
Reproducibility of photoacoustic imaging measurements over time using the quality control phantom. Stability of VisualSonics (A) and Endra (B) over time assessed by repeated imaging of phantom P1 at 750 nm. First 30 days shown for clarity, but coefficient of variation values derived out to 4 months shown in Table 2. Abbreviation VS = VisualSonics.

The reproducibility of the systems over time was assessed by repeated imaging of the quality control phantom. This was performed hourly over a single day (with removal and replacement of the phantom in the imaging system), then repeatedly up to 4 months from the initial measurement. Figure 5 shows the photoacoustic signal normalized to the background for the first 30 days of imaging; linear fits gave slopes of -0.02465 ± 0.02680 per day and 0.02909 ± 0.1556 per day for VisualSonics and Endra respectively. Neither slope was significantly non-zero (p=0.37 and 0.25 respectively) indicating a stable photoacoustic signal over time. The coefficient of variation of these data over the measured time course is displayed in Table 2.

**Table 2:** Coefficient of variation (COV; %) of the two systems over time.

**% COV**	**VisualSonics**	**Endra**
**COV 1 Day**	6.8	15.3
**COV 1 Month**	13.3	13.5
**COV 4 Months**	13.5	13.8

This calculation was made using the mean target signal in the quality control phantom (P1 in Table 1) over different durations of time.

### 3.2: Optimization of photoacoustic imaging parameters for maximum signal-to-noise ratio using the quality control phantom

**Figure 6:**
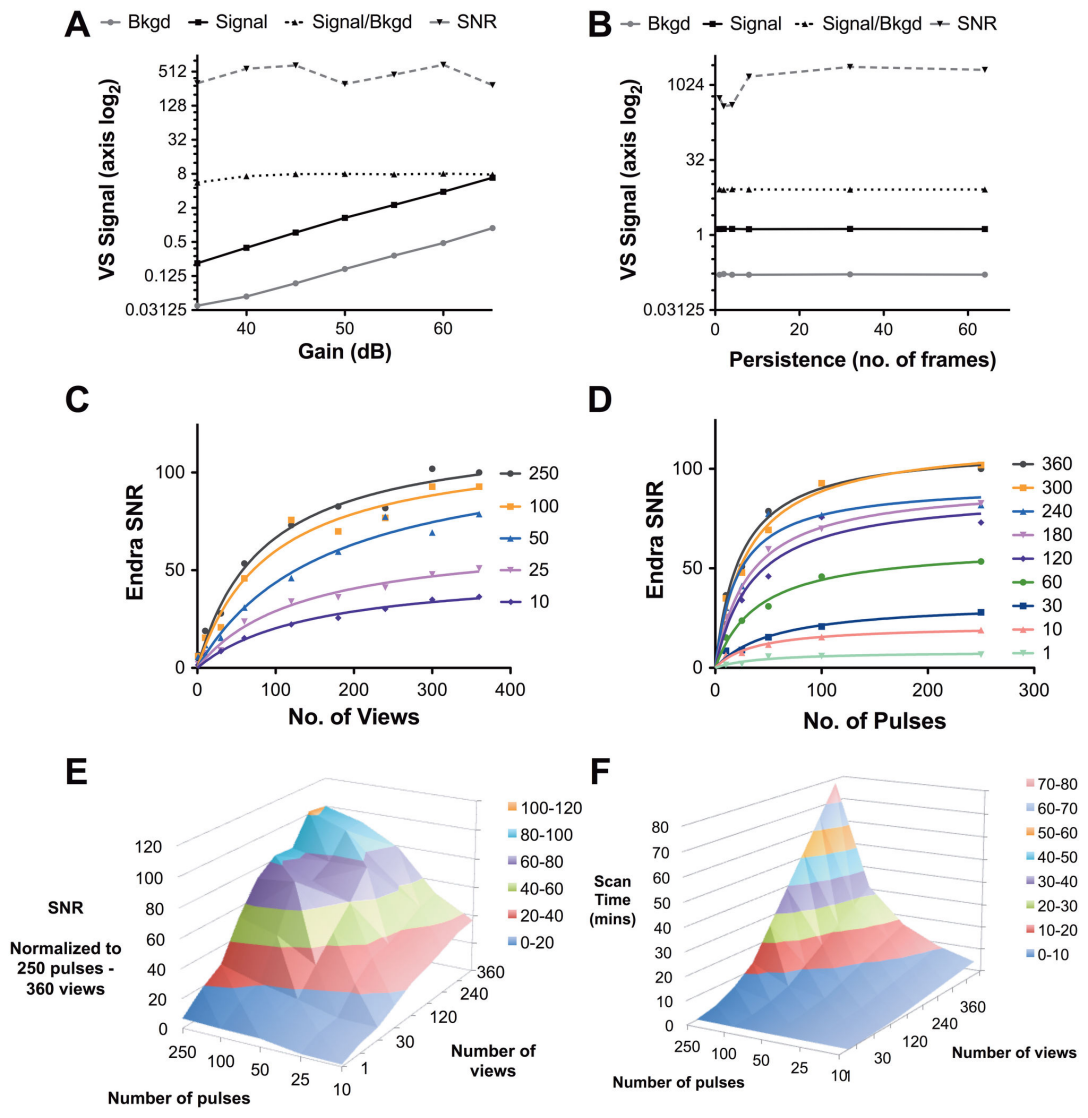
Verification of changing system settings and their effect on photoacoustic imaging signals. Data is shown for each instrument derived from imaging the quality control phantom as a function of wavelength. The effects of changing VisualSonics Vevo LAZR gain and persistence (signal averaging) on the photoacoustic signals from the target sphere are illustrated in A and B. Increasing the number of angular views (C) and pulses (D) increases the signal-to-noise ratio (SNR) logarithmically on the Endra system. The combined effect is illustrated in (E), while the resulting increase in scan time is illustrated in (F). All Endra SNR data is normalized to the SNR measured in the image volume acquired with 250 pulses and 360 views. Abbreviation VS = VisualSonics.

The signal-to-noise ratio (SNR) in photoacoustic images is primarily determined by the absorbed optical energy in the target, which will depend on the energy and number of laser pulses used to produce the image. Other system settings can be used to improve the SNR when the energy absorption is fixed. Images from phantom P1 (see Table 1) were used for this study. For the VisualSonics system, imaging parameters tested include gain, persistence and B-mode focus. As expected, increasing the gain setting (in decibels, dB) increased the photoacoustic signals from both the spherical target and the background, but had no effect on either the signal-to-background ratio (SBR) or SNR (Figure 6A). Increasing persistence, a pixel averaging operation that operates on successive frames acquired by the system, improves image SNR when 8 or more frames are used for averaging (Figure 6B). The transducer focus set for the concurrent B-Mode imaging does not affect the photoacoustic signal output (data not shown). Based on the results of these studies, the gain was set to 52 dB, persistence to 8 and B-mode focus at 10 mm in all other imaging studies unless otherwise stated.

For the Endra system, the trade-off between scan time, number of angular views (improving resolution of features in reconstructed images) and number of averaged laser pulses (improving sensitivity) is important for small animal imaging. The P1 phantom was scanned with the number of laser pulses between 10 and 250, and views between 1 and 360. The scan times ranged from ~ 10 s for 1 pulse at 10 views, to 78 minutes for 250 pulses at 360 views. The SNR shows a logarithmic dependence on increasing views (for a fixed number of pulses, Figure 6C) and pulses (for a fixed number of views, Figure 6D) and this is further illustrated in Figure 6E. These results can be directly compared with the increase in scan time shown in Figure 6F. Unless otherwise stated, all other experiments were performed with 120 angular views and 50 laser pulses at 750 nm, representing an acceptable compromise between SNR and scan time (6 minutes).

### 3.3: Effect of optical background on the detection of photoacoustic signals measured with the robustness phantoms

**Figure 7:**
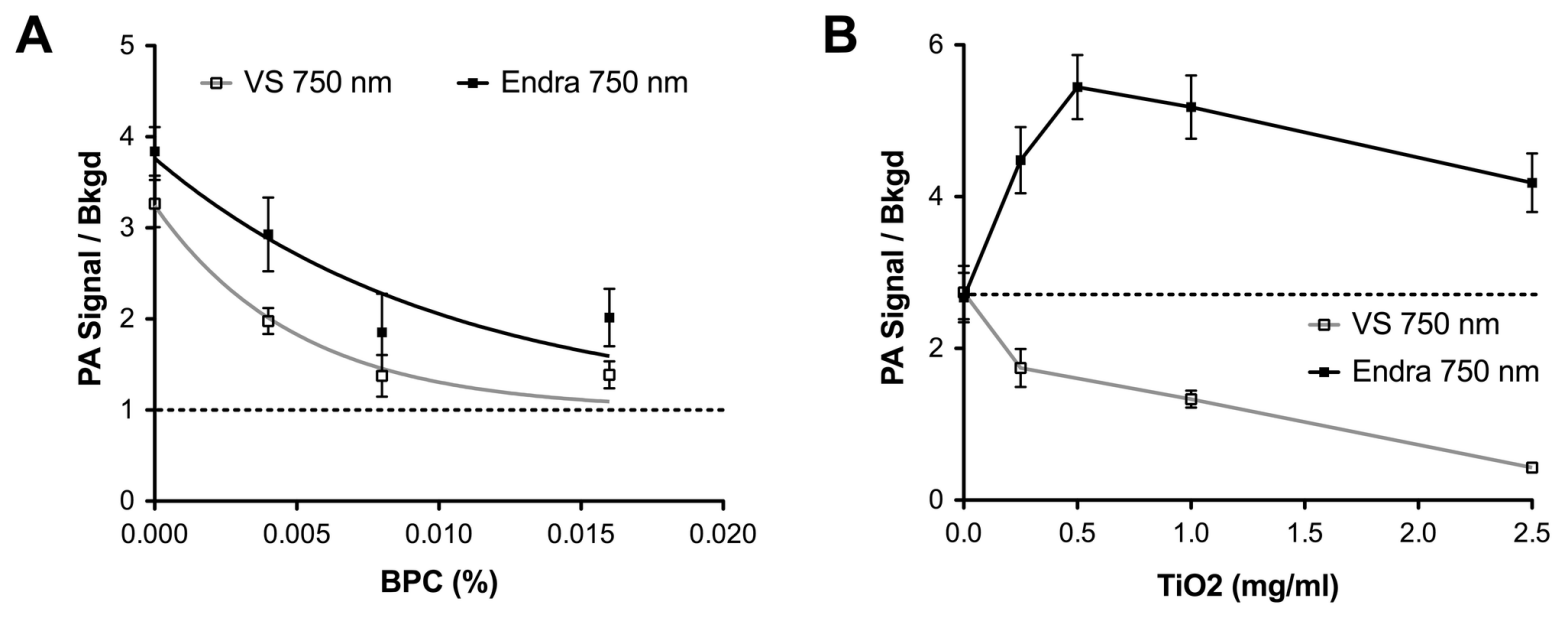
Detection limits for photoacoustic signals from target spheres in the presence of optically absorbing (A) or scattering (B) background PVCP. Phantoms of the ranges P2 (A) and P3 (B) (see Table 1) were used to perform the measurements. Data in (A) were fit to a monoexponential decay with a plateau of 1. For clarity, data is shown only from the 0.128% BPC sphere at 750 nm; the same trend was observed for all BPC and TiO_2_ concentrations, and wavelengths tested. Abbreviation VS = VisualSonics.

Two sets of phantoms were designed to test the effect of optically absorbing (P2; four phantoms with BPC concentration 0-0.016% v/v) and scattering (P3; four phantoms with TiO_2_ concentration 0-2.5 mg/mL) backgrounds on the detectability of spherical targets with a range of BPC concentrations (0.064-0.256% v/v). These measurements help us to understand the performance limits of the two systems for small animal imaging, by including biologically relevant optical absorption and scattering. The maximum target concentration investigated here mimics hemoglobin at 750 nm [33], while the background absorption and scattering concentration ranges were selected based on reported average values for soft tissue [10]. These phantoms were all imaged at 680, 750, 850 and 950 nm on both instruments; the data shown in Figure 7 is from a spherical target with 0.128% v/v BPC in a background concentration range of 0 to 0.016% v/v at 750 nm, but the trends observed were similar for all target concentrations and wavelengths.

The signal-to-background ratio (abbreviated as SBR) falls exponentially as expected with increasing background absorption (Figure 7A, r ^2^ of fit 0.96 and 0.92 for VisualSonics and Endra respectively). Interestingly, while the VisualSonics data indicated a similar exponential decay trend for increased background scattering (r^2^ = 0.94), the Endra data exhibited an initial increase in SBR up to a concentration of 0.5-1 mg/ml TiO_2_, before falling off towards SBR = 1 (Figure 7B). This observation could be explained by a number of factors, which are described in the Discussion section below. In both cases, the PA signal measured in the background regions of P2 phantoms was linear with increasing BPC concentration. Black plastic color (BPC) at concentrations as low as 0.004% (volume fraction; v/v, *μ*_*a*_= 0.025 ± 0.01 cm^-1^) within polyvinyl chloride plastisol (PVCP) phantoms resulted in readily detectable photoacoustic signals on both of the small animal imaging systems. The signal from the spherical targets was also linear with increasing BPC concentration within the target, independent of the optical absorption or scattering of the background matrix. The PA signal recorded with both instruments in the background of the P3 (TiO_2_) phantoms at the same depths as the targets showed a slight increase (~10%) from 0 to 2.5 mg/ml, but this was not significant.

### 3.4: Imaging depth of penetration assessed using the depth phantoms

**Figure 8:**
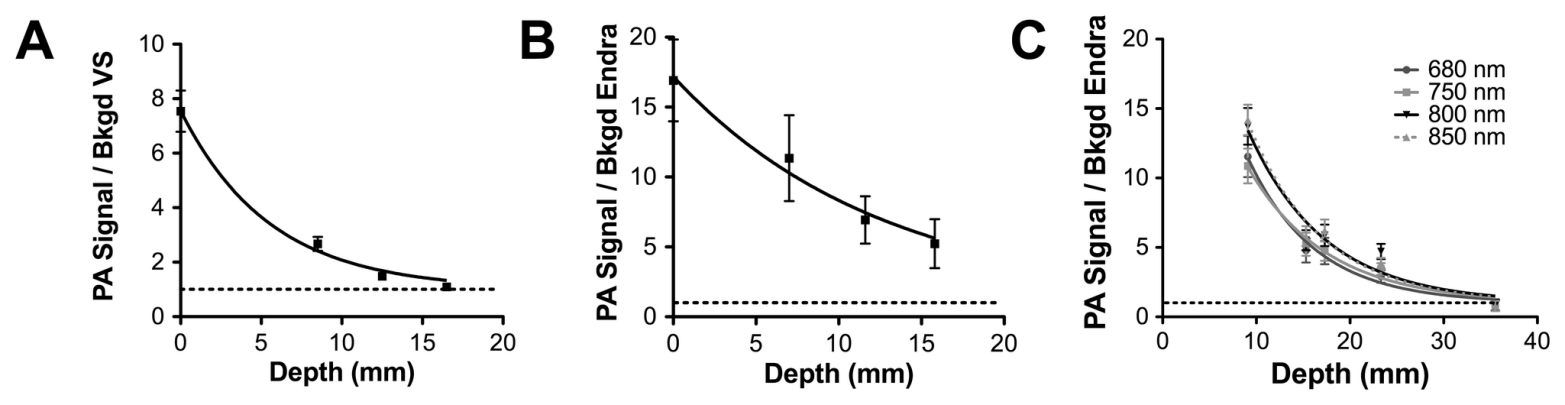
Detection limits as a function of depth in optically absorbing and scattering background PVCP (0.004% BPC and 0.5 mg/ml TiO_2_; 0.256% BPC target). Data in A and B are images from phantom P4 acquired by VisualSonics and Endra respectively and were fit to a monoexponential decay function with a plateau of 1. As the Endra signal had yet to plateau, the second phantom P5 was used to push the limits of detection and this was imaged at multiple wavelengths as shown in (C). Abbreviation VS = VisualSonics.

Two different phantoms were constructed to assess the change in photoacoustic signal from the spherical targets as a function of depth (phantom P4 and P5). The effect of depth was first explored using phantom P4, data from which is shown in Figure 8A and 8B, with the curve fit a monoexponential decay (plateau = 1, r^2^ = 0.98 and 0.99). With VisualSonics (Figure 8A), an SBR of ~1 was observed at the maximum target depth used in phantom P4 (16 mm); this was not the case with the Endra system (Figure 8B). An additional phantom, P5, was used to further explore the depth of penetration possible with the Endra system as a function of target BPC concentration and imaging wavelength, within a fixed background. As can be seen from Figure 8C, an SBR of ~1 was found at a depth of 35.5 mm. The lowest SBR is observed at 680 nm and this increases up to 850 nm, as expected due to the increased penetration of the light at longer wavelengths.

## Discussion

Photoacoustic imaging is an exciting new technique for imaging of small animals and has tremendous potential for clinical translation. The lack of a robust and reliable method for quantifying the performance of different photoacoustic imaging instruments before embarking on animal or human studies is a major deficiency in the field. Phantom studies represent a particular challenge for photoacoustic imaging, as the phantom must possess both optical and acoustic properties commensurate with that of soft tissue.

The design and fabrication of physical phantoms from polyvinyl chloride plastisol (PVCP) described in this work has the potential to overcome the present deficiency by providing: a stable, low cost phantom recipe that is easy to prepare; the ability to produce both a background matrix and targets of defined shape and size with the same material; acoustic properties (including density and acoustic parameters) that are similar to those of water; and optical absorbing and scattering properties that can be tailored with additives.

In addition to presenting the design and construction of the phantoms, we also applied the fabricated phantoms to understand the performance limits of two commercial photoacoustic imaging systems designed specifically for preclinical small animal imaging: the VisualSonics Vevo LAZR and Endra Nexus 128. Our quality control phantom allowed us to verify that the data reconstruction techniques were correctly implemented, by the recovery in the image of a spherical absorbing object. We did, however, identify an error in the built-in energy compensation for one of the systems, which we rectified by applying a manual energy correction. We then found that both small animal imaging systems gave accurate “flat” spectral responses to the BPC dye, once the appropriate energy compensation was performed, and also produced photoacoustic signals that were linear with the concentration of dye. These photoacoustic signals were reproducible as a function of time over a period of months, with the coefficient of variation in repeated phantom imaging being less than 15% over 4 months in both cases. We also used the quality control phantom to establish appropriate imaging parameters with which to maximize SNR within a given scan; these parameters were used for the rest of the imaging experiments. The quality control phantoms are stored with the imaging instruments and are routinely imaged to maintain a log of system performance.

Our phantom studies raised some interesting differences between the performance of the two systems, particularly with regard to imaging at depth in the presence of an optically scattering background. Based on the behavior observed when imaging our robustness phantom, a small amount of scattering material appeared to increase the detected PA signal for phantoms placed in the Endra system, before an excess reduced it, while in the VisualSonics system, optical scattering appeared to reduce the PA signal detected from targets embedded at 5 mm below the surface of the phantom. Furthermore, measurements of the depth phantom showed that the depth of penetration achievable for the Endra system for targets embedded in a mildly absorbing and scattering background matrix was more than double that of the VisualSonics system.

A combination of the different laser illumination, transducer frequencies and acquisition geometries could explain these observations. The Endra system employs diffuse laser light to excite photoacoustic signals in ~ 20 mm diameter field of view so increasing the optical scattering background could more uniformly distribute light within the phantom volume. The crossed laser beam geometry of the VisualSonics system puts both illumination and acoustic detection along a vertical plane; therefore optical scattering could direct light away from the plane of maximum sensitivity. However, as light propagates diffusively beyond the transport mean free path (~ 1-2 mm), it is more likely that the difference in acoustic acquisition better explains this observation. The higher center frequency of the VisualSonics system (21 MHz vs. 5 MHz Endra) could reduce the depth of photoacoustic signal detection, since higher frequency acoustic waves are attenuated to a greater extent. Finally and perhaps most importantly, transducers in the Endra scanner are distributed on a hemispherical bowl for tomographic acquisition, rather than along a line for the VisualSonics planar acquisition, detecting acoustic waves emitted over a greater solid angle. A small increase in optical scattering may therefore increase the detected signal as a more uniform light fluence distribution around the target may contribute signals to a higher proportion of the 128 transducers.

Our findings therefore underscore the importance of testing photoacoustic imaging systems using stable physical phantoms before performing *in vivo* measurements. Inaccurate reconstructions or energy compensation could lead to incorrect readouts of endogenous signals, such as oxygen saturation, or affect quantification of injected contrast agent signals. It is also clear that the presence of biologically relevant amounts of absorption and scattering media within the imaging volume can impact the achievable signal-to-noise ratio and will therefore affect the lower limit of quantification of contrast agents as well as the depth of penetration that is available in a given system. These results could inform on future system designs, contribute to the development of light models to improve reconstruction algorithms and help to characterize improvements in the next generation of these instruments.

Using the same phantoms to test both systems enabled a direct comparison of their performance. The two systems evaluated here demonstrated excellent reproducibility, linearity and penetration depth for preclinical small animal photoacoustic imaging. The increased imaging depth and sensitivity of the tomographic Endra images, which enable visualization of deep tissue structures, come at the expense of longer scan times of around 6 minutes per scan in this study. Although the geometry of the VisualSonics system may result in a lower photoacoustic imaging sensitivity and depth in soft tissue, the depth of penetration is more than enough to image subcutaneous tumors and superficial organs. Moreover, the VisualSonics system provides a significant advantage in terms of acquisition speed, with a typical frame rate of 5 Hz for a single planar view, providing the user with real time feedback as well as ultrasound co-registration and a software package in which imaging and analysis can be performed simultaneously.

Additional studies will be needed to establish PVCP more generally as a standard photoacoustic imaging phantom material. We selected a range of *μ*_*a*_ and $\mu_{S}^{'}$ based on literature values averaged over a range of tissue types and wavelength [10,24,25] to define the range of BPC dye and titanium dioxide concentrations used in this work. When the imaging system is solely for application with a particular biological tissue e.g. prostate [5,34], the absorbing and scattering properties of characterization phantoms should be tailored to the values for that tissue to ensure that the imaging performance is specifically optimized for the application. The acoustic properties of PVCP, which have been established previously [20], were not explored here as our study was focused on developing a stable phantom for quality control and system evaluation. Changing the ultrasound properties of PVCP using plastic hardener and acoustic scatterers, in order to modulate the speed of sound, ultrasound attenuation and backscatter, is the subject of ongoing work in our laboratory. The combination of both optical and ultrasound modulation within the bounds of the soft tissue averages will be necessary to establish the photoacoustic detection limits for a given imaging application.

In summary, we have shown that PVCP provides a stable, low cost, material for creating stable physical phantoms suitable for exploring the performance of new photoacoustic imaging systems.

## Supporting Information

Protocol S1 (DOCX)
