# Supplementary material for: Development and Application of Stable Phantoms for the Evaluation of Photoacoustic Imaging Instruments
Source: PLoS One. 2013 Sep 25;8(9):e75533. doi: 10.1371/journal.pone.0075533 (PMC3783368; doi:10.1371/journal.pone.0075533)
Supplement: Protocol S1 — (DOCX) [file pone.0075533.s001.docx]

**Supplementary Methods**

**Development and Application of Stable Phantoms for the Evaluation of Photoacoustic Imaging Instruments**

Sarah E. Bohndiek, Sandhya Bodapati, Dominique Van De Sompel, Sri-Rajasekhar Kothapalli and Sanjiv S. Gambhir

Affiliation of all authors: Bio-X Program and Department of Radiology, Molecular Imaging Program at Stanford, Stanford University School of Medicine, Stanford, CA, 94305

**Protocol S1: Procedure for developing PVCP phantoms**

The following materials are required for phantom preparation:

- Polyvinyl Chloride Plastisol (PVCP; M-F Manufacturing Co., Fort Worth, TX, USA)
- Black Plastic Color (BPC; M-F Manufacturing Co., Fort Worth, TX, USA)
- Titanium Oxide (TiO_2_) powder (232033; Sigma-Aldrich, St. Louis, MO, USA)
- Mold (in this case, the mold was manufactured in-house by Varian Physics Workshop, Stanford University)
  - The mold should be resistant to temperatures over 180 °C
  - Appropriate materials for making a mold for the background matrix are acrylic and aluminum
  - For making spherical targets, we suggest aluminum to allow uniform heating of the mold prior to pouring PVCP (see below)
- Heat proof gloves (Clavies Autoclave Gloves Z408492; Sigma-Aldrich, St. Louis, MO, USA)
- C clamp (Kant-Twist Clamp 2”; Newman Tools Inc., Stittsville, ON, Canada)
- 100 mL round bottom flask
- Oil bath and heated stirrer plate with stirrer bar
- Vacuum line
- Fume hood

Optical absorber (BPC) and scatterer (TiO_2_) should be added to liquid PVCP at the desired concentration and sonicated at 40 °C for 10 minutes immediately prior to beginning the procedure below, in order to ensure uniform distribution of the solutes. For linearity measurements, a serial dilution from the most concentrated solution provides the most accurate results.

*Procedure:*

1. Turn on heated stirrer plate (in fume hood) with set point at 200 °C and oil bath on top and wait for it to reach the set point
2. Pour 20 mL PVCP mixture into 100 mL capacity round bottom flask and add a magnetic stirrer bar
   1. The suggested volume can be used to make phantoms for small animal imaging systems. Larger volumes can be prepared in a proportionately larger vessel (volumes of up to 300 mL can be produced if needed). Uniform heating is important to ensure a reproducible phase transition.
3. Connect the flask to a vacuum line (with a valve)
4. Gradually open the vacuum line until small bubbles are observed in the PVCP mixture.
   1. You may need to adjust the vacuum throughout the heating procedure to avoid excessive bubbling
5. Place the RBF into the oil bath and begin stirring. Start a timer.
6. After 6 minutes, the phase transition should begin and the material will become viscous (temperature around 130 °C); reduce the stirring speed
7. At 8 minutes, the transition should be nearing completion and the material will begin to move more freely; increase stirring speed again back to the original value
8. After 10 minutes, the transition should be complete and the stirrer bar should be moving freely
   1. The temperature of the PVCP at this stage is usually close to 180 °C
   2. The timing of the transition will be longer for larger volumes. Use the change in viscosity to assess the required time before making phantoms.
9. Release the vacuum
10. Remove the flask from the oil bath
11. Immediately pour the PVCP into a heat proof mold
    1. Lubricate the surface of the mold to allow easy removal of the phantom if needed
    2. To make spheres, preheat the aluminum mold to 50 – 60 °C then handle with heat proof gloves. Lubricate the aluminum surface lightly to allow easy removal of spheres. Use a wide spatula to guide the liquid over the surface then rapidly and tightly clamp the two sides of the mold together using a C clamp. Allow to cool for at least 10 minutes before opening.
    3. When creating layered phantoms, pour the first layer and allow to cool for 5 – 10 seconds before adding spheres at the required positions using forceps. Repeat the PVCP preparation procedure for each layer.
    4. PVCP remains liquid until it reaches around 140 °C
    5. Do not reheat otherwise a color change will be observed
12. Allow to set for a few minutes then the phantom will be ready to use
    1. Larger phantoms will require a longer curing time.
